# Effects of Altered Levels of Pro- and Anti-Inflammatory Mediators on Locations of In-Stent Reocclusions in Elderly Patients

**DOI:** 10.1155/2020/1719279

**Published:** 2020-09-23

**Authors:** Xia Li, Dianxuan Guo, Ying Chen, Youdong Hu, Fenglin Zhang

**Affiliations:** Department of Geriatrics, The Affiliated Huaian Hospital of Xuzhou Medical University, Huaian 223002, China

## Abstract

Imbalances of proatherogenic inflammatory and antiatherogenic inflammatory mediators were involved in the pathogenesis of atherosclerosis. This study sought to investigate the effects of proatherogenic inflammatory and antiatherogenic inflammatory mediators on the proximal, middle, and distal coronary artery reocclusions in elderly patients after coronary stent implantations. We measured the expression levels of proatherogenic inflammatory/antiatherogenic inflammatory cytokines. This included interleukin-1 *β* (IL-1 *β*), interleukin-6 (IL-6), interleukin-8 (IL-8), tumor necrosis factor-*α* (TNF-*α*), high-sensitivity C-reactive protein (hs-CRP), interleukin-10 (IL-10), interleukin-17 (IL-17), interleukin-13 (IL-13), and interleukin-37 (IL-37) in the elderly patients with the proximal, middle, and distal coronary artery reocclusions after coronary stent implantations. Levels of IL-1 *β*, IL-6, IL-8, TNF-*α*, and hs-CRP were remarkably increased (*P* < 0.001), and levels of IL-10, IL-17, IL-13, and IL-37 were remarkably lowered (*P* < 0.001) in the elderly patients with the proximal, middle, and distal coronary artery reocclusions. Imbalances of proatherogenic inflammatory and antiatherogenic inflammatory mediators may be involved in the formation and progression of proximal, middle, and distal coronary artery reocclusions in elderly patients after coronary stent implantations.

## 1. Introduction

The cytokines were related to the complex proinflammatory responses in the atherosclerotic lesions, and the cytokines were involved in the formation and disruption of atherosclerotic plaques. The cytokines were divided into proatherogenic inflammatory and antiatherogenic inflammatory cytokines depending on whether they led to the development and progression of atherosclerosis [1].

Atherosclerosis was a chronic inflammatory disease, and the proatherogenic inflammatory cytokine interleukin-1 *β* (IL-1 *β*) was related to the progression of atherosclerosis and the lack of IL-1 *β* reduced the development of atherosclerosis [2, 3]. The proatherogenic inflammatory cytokine interleukin-6 (IL-6) was a biomarker of the blood vascular inflammation that was an important risk factor for cardiovascular diseases. The IL-6 played a key role in inflammatory response and atherosclerosis [4]. The interleukin-8 (IL-8) expression was in human atherosclerotic lesions, and increased levels of proatherogenic inflammatory cytokine IL-8 were related to the development of atherosclerosis, and IL-8 was involved in the unstable coronary heart disease [5, 6]. Proatherogenic inflammatory cytokine tumor necrosis factor-*α* (TNF-*α*) was elevated in patients with unstable atherosclerotic lesions and acute myocardial infarction. Increased levels of TNF-*α* were the predictions of sudden coronary death and cardiovascular events after acute myocardial infarction. The TNF-*α* was related to severe atherosclerotic plaques and played a key role in the development of coronary heart disease [7, 8]. High-sensitivity C-reactive protein (hs-CRP) was the inflammatory biomarker, synthesizing through the liver under stress, and was involved in the inflammatory response. The levels of hs-CRP were elevated in patients with acute coronary syndrome undergoing percutaneous coronary intervention [9].

Interleukin-10 (IL-10) was an antiatherogenic inflammatory cytokine that played the protective role in coronary atherosclerosis, and IL-10 deletion accelerated the progression of atherosclerosis. IL-10 decreased atherogenesis and enhanced the stability of coronary plaques [10]. The administration of inhibitors of the interleukin-17 (IL-17) signaling pathway and decreased IL-17 as antiatherogenic inflammatory cytokine was related to the high risk of major adverse cardiac events in the patients with an acute heart attack. High levels of IL-17 were related to the decreased atherosclerotic plaque size and artery plaque stability. The specific deletion of the IL-17 signaling pathway inhibited the atheroprotective effect and led to vascular inflammatory response and promoted the development of atherosclerosis [11]. The antiatherogenic inflammatory cytokine interleukin-13 (IL-13) had an anti-inflammatory response and the antiatherogenic role by inhibiting atherosclerotic plaque compositions and modulating the morphology of atherosclerotic plaques, and the deletion of IL-13 accelerated the formation of atherosclerosis. IL-13 also inhibited proatherogenic inflammatory cytokines (TNF-*α* and IL-1 *β*) [12, 13]. The overexpression of interleukin-37 (IL-37) as antiatherogenic inflammatory cytokine is protected against the inflammatory response by inhibiting proinflammatory cytokines [14, 15], and IL-37 inhibited atherosclerosis and was a novel cytokine for the prevention and treatment of atherosclerotic diseases [16]. In the present study, we tried to measure the levels of proatherogenic inflammatory and antiatherogenic inflammatory mediators in the elderly patients with proximal, middle, and distal coronary artery reocclusions after coronary stent implantations and to clarify whether imbalances of proatherogenic inflammatory and antiatherogenic inflammatory mediators were involved in the formation and progression of proximal, middle, and distal coronary artery reocclusions after coronary stent implantations in the elderly patients.

## 2. Materials and Methods

### 2.1. Patient Population

From 3rd of April 2013 to 8th of March 2017, this study included consecutive patients with distal coronary artery (Dist CA) reocclusion (*n* = 165), middle coronary artery (Mid CA) reocclusion (*n* = 147), proximal coronary artery (Prox CA) reocclusion (*n* = 123), and proximal+middle coronary artery (Prox Mid CA) reocclusion (*n* = 101). The inclusion criteria in this research project were (1) the patients aged 65 to 88 years old, (2) the patients with proximal, middle, and distal coronary artery reocclusions after coronary stent implantations. The research protocols were approved by the Xuzhou Medical University and the Research Ethics Committee of University Affiliated Huaian Hospital and the Medical Review Board in accordance with relevant laws and regulations of Chinese Government, and all patients gave their written informed consent prior to inclusion into the research protocols in accordance with the Declaration of Helsinki. The following conditions were excluded from the research participation: (1) the uses of proatherogenic inflammatory cytokine antagonists (Beta-adrenoceptor antagonists and Canakinumab), (2) the uses of anti-inflammatory and antiatherogenic agents (Hydrocortisone, Dexamethasone, 1,25 Dihydroxyvitamin D3 and Tanderil), (3) rheumatic diseases, (4) inflammatory bowel diseases, (5) neurodegenerative diseases, (6) infectious diseases, (7) multiple sclerosis, (8) arthritis, (9) asthma, (10) acute respiratory distress syndrome, (11) bronchiectasis, (12) Behçet's disease, (13) periodontal disease, (14) peripheral arterial and veno-occlusive diseases, (15) autoimmune diseases, and (16) acute coronary reocclusion.

### 2.2. Study Design

Health individuals were divided into a control (CON) group (*n* = 69). The patients with right coronary artery reocclusion were divided into distal right coronary artery (Dist RCA) reocclusion group (*n* = 60), middle right coronary artery (Mid RCA) reocclusion group (*n* = 53), proximal right coronary artery (Prox RCA) reocclusion group (*n* = 49), and proximal+middle right coronary artery (Prox Mid RCA) reocclusion group (*n* = 40). The patients with left circumflex artery reocclusion were divided into distal left circumflex artery (Dist LCX) reocclusion group (*n* = 50), middle left circumflex artery (Mid LCX) reocclusion group (*n* = 45), proximal left circumflex artery (Prox LCX) reocclusion group (*n* = 39), and proximal+middle left circumflex artery (Prox Mid LCX) reocclusion group (*n* = 32). The patients with left anterior descending artery reocclusion were divided into distal left anterior descending artery (Dist LAD) reocclusion group (*n* = 55), middle left anterior descending artery (Mid LAD) reocclusion group (*n* = 49), proximal left anterior descending artery (Prox LAD) reocclusion group (*n* = 35), and proximal+middle left anterior descending artery (Prox Mid LAD) reocclusion group (*n* = 29).

### 2.3. Quantitative Evaluations of the Cardiac Angiographies and Echocardiograms

The proximal, middle, and distal coronary artery reocclusions were performed using the cardiovascular angiographic analysis system (QAngio XA). The coronary artery angiograms were confirmed independently by two prominent cardiologists blinded the patient's clinical data. The Doppler echocardiograms were performed with synchronized electrocardiography recordings by independent cardiac medicine experts blinded to the clinical data in accordance with the standards committee of the American Society of Echocardiography.

### 2.4. Determinations of the Levels of IL-1 *β*, IL-6, IL-8, TNF-*α*, and hs-CRP

For analyses of IL-1 *β*, IL-6, IL-8, and TNF-*α*, the blood samples were collected from the patients at 8 am after an overnight fast. The serum samples were immediately centrifuged and were stored at -80°C until further determination. IL-1*β*, IL-6, and IL-8 were determined with the commercial enzyme-linked immunosorbent assay kits (human IL-1*β*: R&D Systems INC., Minnesota, USA; human IL-6: high sensitivity enzyme-linked immunosorbent assay, Immunotech S. A, Marseille, France; human IL-8: enzyme-linked immunosorbent assay; BD Bioscience, La Jolla, CA) as previously described [17, 18]. The serum samples were stored in -80°C until further determination, and the serum levels of TNF-*α* were determined by a high sensitivity enzyme-linked immunosorbent assay kit (Biosource, International, Inc.) according to the manufacturer's instructions [19]. The serum levels of high sensitive C reaction protein (hs-CRP) were analyzed with a commercially available assay kit (hs-CRP test kits, Millipore Corp, Billerica, MA, USA), and the levels of hs-CRP were expressed as mg/L.

### 2.5. Determinations of the Levels of IL-10, IL-17, IL-13, and IL-37

Blood samples from patients were collected at 8 am after 12 hours fast for the determinations of IL-10, IL-17, IL-13, and IL-37. The blood serum samples were stored at -80°C for this study until further determination. Serum levels of IL-10, IL-17, IL-13, and IL-37 were tested with the enzyme-linked immunosorbent assays (IL-10: R&D Systems INC., Minnesota, USA; IL-17: R&D Systems, Minneapolis, MN, USA; IL-13: Calbiochem, San Diego, CA, U.S.A.; IL-37: R&D Systems INC, Minnesota, USA) according to the manufacturer's instructions [11, 13, 16, 20].

### 2.6. The Related Data in Type, Length, and Diameter of Stents

The patients underwent percutaneous coronary intervention for coronary reocclusions with drug-eluting stent implantations before this study. In our research center, the sirolimus-eluting stents (Cordis, Warren, New Jersey, USA) were available in diameters of 2.50 mm, 3.00 mm, 3.50 mm, and 4.00 mm. The stent length was 8 mm for stents with a diameter of 2.50 mm, 15 mm for stents with a diameter of 3.00 mm, 18 mm for stents with a diameter of 3.50 mm, and 23 mm for stents with a diameter of 4.00 mm.

### 2.7. Myocardial Ischemia Flow Grades and Angina Pectoris Grades

This study included the patients with coronary in-stent chronic total reocclusion defined as total coronary artery reocclusion of ≥3-month duration with coronary artery 100% diameter restenosis and thrombolysis in myocardial ischemia (TIMI) flow grade 0 in the target artery after successful coronary stenting. Angiographic TIMI flow grade is used for the assessment of myocardial ischemia degree in the patients according to grade 0 defined as no flow, grade 1 defined as penetration without perfusion, grade 2 defined as partial perfusion, and grade 3 defined as complete perfusion of flow.

Angina pectoris, the pain of myocardial ischemia, is the main clinical symptom of coronary in-stent chronic total reocclusion. In this study, the stable angina pectoris (SAP) is classified into class I defined as no limitation in ordinary physical activities, class II defined as slight limitation of ordinary activities, and class III defined as marked limitation of ordinary activities according to the Canadian Cardiovascular Society Classification. The unstable angina pectoris (UAP) is classified into grade-I defined as accelerated angina pectoris, grade-II defined as subacute angina at rest, and grade-III defined as acute angina at rest according to Braunwald Classification.

### 2.8. Determinations of TC, TG, HDL-C, LDL-C, and VLDL-C

All patients received continuative treatment with atorvastatin 20 mg/d after coronary stent implantation. Fasting blood samples were taken from all patients in the morning. The serum levels of total cholesterol (TC), triglyceride (TG), high-density lipoprotein cholesterol (HDL-C), low-density lipoprotein-cholesterol (LDL-C), and very low-density lipoprotein-cholesterol (VLDL-C) were measured by HLC-729LPII (Tosoh Corporation).

### 2.9. Statistical Analysis

For all these experiments, all quantitative research data on the proatherogenic inflammatory/antiatherogenic inflammatory cytokine levels were expressed as the mean ± standard deviation (mean ± SD). The paired two-sample Student's *t*-tests were used for assessing every pair of the matching observed data, and the one-way analysis of variance was performed to compare the variance among the means of different groups. Multivariate regression analysis was used for evaluating the significance of variables for the proximal, middle, and distal coronary artery reocclusions. The observed *P* values smaller than 0.05 (*P* < 0.05) were considered to indicate a statistically significant difference. All statistical analyses were conducted using the SPSS statistical package version 20.0 software (IBM Corp., Armonk, NY, USA) for all statistical tests of differential expression of proatherogenic inflammatory and antiatherogenic inflammatory mediators.

## 3. Results

### 3.1. Basic Clinical Features of the Elderly Patients with the Proximal, Middle, and Distal Coronary Artery Reocclusions

The baseline clinical characteristics were very similar among the different study groups of the elderly patients with the proximal, middle, and distal coronary artery reocclusions (Table 1). The patients in each group were well matched without statistically differences in gender (*P* = 0.14), age (*P* = 0.57), familial coronary artery disease defined as asymptomatic persons with a parent or sibling with coronary artery disease (*P* = 0.49), chest pain defined as pain located in the chest region (*P* = 0.12), alcohol consumption defined as 1-2 drinks/day about 3 times weekly (*P* = 0.08), smoking defined as a person who smokes tobacco regularly (*P* = 0.50), dyslipidaemia defined as a condition marked by abnormal concentrations of lipids or lipoproteins in the blood (*P* = 0.09), diabetes mellitus defined as the fasting plasma glucose ≥ 126 mg/dL (7.0 mmol/L); or 2-h plasma glucose ≥ 200 mg/dL (11.1 mmol/L) during oral glucose tolerance test; or hemoglobin A1c ≥ 6.5% (48 mmol/mol) (*P* = 0.41), and hypertension defined as systolic blood pressure ≥ 140 mm Hg, or diastolic blood pressure ≥ 90 mm Hg (*P* = 0.10).

### 3.2. Cytokine and hs-CRP Levels in the Elderly Patients with the Proximal, Middle, and Distal CA Reocclusions

The levels of IL-1 *β*, IL-6, IL-8, TNF-*α*, and hs-CRP were increased significantly in Prox CA reocclusion group compared to Mid CA reocclusion and Dist CA reocclusion groups, respectively (*P* < 0.001) and were further increased significantly in Prox+Mid CA reocclusion group compared to Prox CA reocclusion and Mid CA reocclusion groups, respectively (*P* < 0.001). The levels of IL-10, IL-17, IL-13, and IL-37 were decreased significantly in Prox CA reocclusion group compared to Mid CA reocclusion and Dist CA reocclusion groups, respectively (*P* < 0.001) and were further decreased significantly in Prox+Mid CA reocclusion group compared to Prox CA reocclusion and Mid CA reocclusion groups, respectively (*P* < 0.001). These data suggested that increased levels of IL-1 *β*, IL-6, IL-8, TNF-*α*, and hs-CRP and decreased levels of IL-10, IL-17, IL-13, and IL-37 promoted the proximal, middle, and distal CA reocclusions in the elderly patients after coronary artery implantations. Imbalances of proatherogenic inflammatory and antiatherogenic inflammatory cytokines were involved in the proximal, middle, and distal CA reocclusions in the elderly patients after coronary artery implantations (Table 2).

### 3.3. Levels of IL-1 *β*, IL-6, IL-8, TNF-*α*, IL-10, hs-CRP, IL-17, IL-13, and IL-37 in the Elderly Patients with the Proximal, Middle, and Distal RCA Reocclusions

The levels of IL-1 *β*, IL-6, IL-8, TNF-*α*, and hs-CRP were increased significantly in Prox RCA reocclusion group compared to Mid RCA reocclusion and Dist RCA reocclusion groups, respectively (*P* < 0.001) and were further increased significantly in Prox+Mid RCA reocclusion group compared to Prox RCA reocclusion and Mid RCA reocclusion groups, respectively (*P* < 0.001). The levels of IL-10, IL-17, IL-13, and IL-37 were decreased significantly in Prox RCA reocclusion group compared to Mid RCA reocclusion and Dist RCA reocclusion groups, respectively (*P* < 0.001), and were further decreased significantly in Prox+Mid RCA reocclusion group compared to Prox RCA reocclusion and Mid RCA reocclusion groups, respectively (*P* < 0.001). These data suggested that increased levels of IL-1 *β*, IL-6, IL-8, TNF-*α*, and hs-CRP and decreased levels of IL-10, IL-17, IL-13, and IL-37 were related to the proximal, middle, and distal RCA reocclusions in the elderly patients after coronary artery implantations. Imbalances of proatherogenic inflammatory and antiatherogenic inflammatory mediators promoted the proximal, middle, and distal RCA reocclusions in the elderly patients after coronary artery implantations (Table 3).

### 3.4. Expressions of IL-1 *β*, IL-6, IL-8, TNF-*α*, hs-CRP, IL-10, IL-17, IL-13, and IL-37 in the Elderly Patients with the Proximal, Middle, and Distal LCX Reocclusions

The levels of IL-1 *β*, IL-6, IL-8, TNF-*α*, and hs-CRP were increased significantly in Prox LCX reocclusion group compared to Mid LCX reocclusion and Dist LCX reocclusion groups, respectively (*P* < 0.001), and were further increased significantly in Prox+Mid LCX reocclusion group compared to Prox LCX reocclusion and Mid LCX reocclusion groups, respectively (*P* < 0.001). The levels of IL-10, IL-17, IL-13, and IL-37 were decreased significantly in Prox LCX reocclusion group compared to Mid LCX reocclusion and Dist LCX reocclusion groups, respectively (*P* < 0.001), and were further decreased significantly in Prox+Mid LCX reocclusion group compared to Prox LCX reocclusion and Mid LCX reocclusion groups, respectively (*P* < 0.001). Imbalances of proatherogenic inflammatory and antiatherogenic inflammatory mediators had a significantly higher risk of developing proximal, middle, and distal LCX reocclusions after coronary artery implantations (Table 4).

### 3.5. Expression Levels of IL-1 *β*, IL-6, IL-8, TNF-*α*, hs-CRP, IL-10, IL-17, IL-13, and IL-37 in the Elderly Patients with the Proximal, Middle, and Distal LAD Reocclusions

The levels of IL-1 *β*, IL-6, IL-8, TNF-*α*, and hs-CRP were increased significantly in Prox LAD reocclusion group compared to Mid LAD reocclusion and Dist LAD reocclusion groups, respectively (*P* < 0.001), and were further increased significantly in Prox+Mid LAD reocclusion group compared to Prox LAD reocclusion and Mid LAD reocclusion groups, respectively (*P* < 0.001). The levels of IL-10, IL-17, IL-13, and IL-37 were decreased significantly in Prox LAD reocclusion group compared to Mid LAD reocclusion and Dist LAD reocclusion groups, respectively (*P* < 0.001), and were further decreased significantly in Prox+Mid LAD reocclusion group compared to Prox LAD reocclusion and Mid LAD reocclusion groups, respectively (*P* < 0.001). The results showed that the imbalances of proatherogenic inflammatory and antiatherogenic inflammatory mediators may accelerate the formation of the proximal, middle, and distal LAD reocclusions after coronary stenting (Table 5).

### 3.6. Stents Features of the Elderly Patients with Distal, Middle, and Proximal Coronary Artery Reocclusions

The type, length, and diameter of stents were very similar among the different study groups of elderly patients with the proximal, middle, and distal coronary artery reocclusions without statistical differences (Table 6).

### 3.7. Data of TIMI Flow Grades and Ischemic Angina Pectoris

The patients with distal, middle, and proximal coronary artery reocclusions in different study groups were well matched without statistical differences in TIMI flow grades, SAP classification, and UAP classification (Table 7).

### 3.8. Levels of TC, TG, HDL-C, LDL-C, and VLDL-C in Elderly Patients with the Proximal, Middle, and Distal CA Reocclusions

The levels TC, TG, HDL-C, LDL-C, and VLDL-C were shown in Table 8.

### 3.9. Multivariate Regression Analysis to Evaluate the Significance of Variables for the Proximal, Middle, and Distal Coronary Artery Reocclusions

By multivariate regression analysis, IL-1 *β*, IL-6, IL-8, TNF-*α*, IL-10, IL-17, IL-13, and IL-37 were found to be independent indicators of the proximal, middle, and distal coronary artery reocclusions after adjustment for age, gender, familial coronary artery disease, chest pain, alcohol consumption, smoking, dyslipidaemia, diabetes mellitus, and hypertension in all patients. A *P* value of less than 0.05 was considered statistically significant (Table 9).

## 4. Discussion

Our study showed that the levels of IL-1 *β*, IL-6, IL-8, TNF-*α*, and hs-CRP as proatherogenic inflammatory mediators were increased significantly in the elderly patients with the proximal, middle, and distal coronary artery reocclusions after coronary stent implantations. These findings suggested that increased levels of IL-1 *β*, IL-6, IL-8. TNF-*α*, and hs-CRP were associated with the proximal, middle, and distal coronary artery reocclusions, and proatherogenic inflammatory mediators were involved in the pathogenesis of the proximal, middle, and distal coronary artery reocclusions in the elderly patients after coronary stent implantations. IL-1 *β* was involved in the progression of atherosclerosis, and the IL-1 *β* deficiency reduced atherosclerotic plaques and inhibited atherosclerosis development [3]. IL-6 was related to atherosclerosis, and high circulating IL-6 levels contributed to foam cell formation in atherosclerotic lesions [4]. IL-8 levels were the strong predictor of coronary heart disease risk and played a key role in the initiation and progression of atherosclerosis [5]. The overexpression of TNF-*α* was an early key event in atherosclerotic lesions and was related to a severe degree of atherosclerosis and thickening of vascular intima [7]. The higher levels of hs-CRP as an inflammatory mediator were related to an increased risk of coronary in-stent restenosis, which made it a potential biomarker of prognosis in coronary in-stent restenosis both on admission and in follow up in patients with coronary heart disease after coronary stent implantations [21]. Our results suggested that the increased levels of IL-1 *β*, IL-6, IL-8, TNF-*α*, and hs-CRP as proatherogenic inflammatory mediators were associated with the proximal, middle, and distal coronary artery reocclusions and the proatherogenic inflammatory mediators may promote the formation of the proximal, middle, and distal coronary artery reocclusions after coronary stent implantations.

The cytokines were divided into two classes (proatherogenic inflammatory and antiatherogenic inflammatory cytokines) depending on whether they contributed or inhibited atherogenesis and coronary artery disease [1]. IL-1 *β* played a key role in proinflammatory mechanisms in the arterial walls and IL-1 *β* as the proatherogenic inflammatory cytokine was involved in the progression of atherosclerosis and the IL-1 *β* deficiency reduced atherosclerotic plaques and inhibited atherosclerosis development [3]. The IL-6 was an important proatherogenic inflammatory cytokine in the pathway toward atherosclerotic events and was related to atherosclerosis and clinical cardiovascular disease risk. High circulating IL-6 levels contributed to foam cell formation in atherosclerotic lesions and were one crucial step in the pathobiology of atherosclerosis [4]. The increased levels of proatherogenic inflammatory cytokine IL-8 were related to the elevated risk of coronary heart disease and cardiovascular events by promoting atherosclerotic plaque instability. IL-8 levels were the strong predictor of coronary heart disease risk and played a key role in the initiation and progression of atherosclerosis [5]. The overexpression of TNF-*α* was an early key event in atherosclerotic lesions and was related to a severe degree of atherosclerosis. TNF-*α* expression promoted the atherosclerosis development and thickening of vascular intima and played a key role in human atherogenesis. The expression of proatherogenic inflammatory cytokine TNF-*α* was continuously increased in the patients with postinfarction and increased an elevated risk of recurrent coronary artery disease events [7]. The inflammatory mediators IL-1 *β*, IL-6, IL-8, and TNF-*α* were the risk factors for coronary in-stent restenosis and predicted the risk of coronary in-stent restenosis after percutaneous coronary intervention [22, 23]. The inflammatory mediators IL-1 *β*, IL-6, IL-8, and TNF-*α* contributed to the coronary local proinflammatory response and participated in the pathogenesis of coronary in-stent restenosis and played the key roles in the pathophysiologic process in the development of coronary in-stent restenosis [24, 25], which ultimately progressed to coronary reocclusion after coronary artery stenting [26]. The levels of the inflammatory mediators IL-1 *β*, IL-6, IL-8, and TNF-*α* were increased with coronary lesion severity (proximal coronary reocclusions combined with diffusely calcified long lesions, middle coronary reocclusions combined with long diffuse lesions, and distal coronary reocclusions combined with short diffuse lesions), and the levels of the inflammatory mediators IL-1 *β*, IL-6, IL-8, and TNF-*α* were positively associated with the degree of occlusive coronary lesions [26]. Therefore, the inflammatory mediators IL-1 *β*, IL-6, IL-8, and TNF-*α* were related to the coronary reocclusion lesions at different locations after percutaneous coronary intervention.

Antiatherogenic inflammatory cytokine IL-10 deficiency elevated atherosclerotic plaque size and promoted the development of atherosclerosis. The human IL-10 gene transfer decreased atherosclerosis and played an important protective role in atherosclerotic plaque stability. Antiatherogenic inflammatory cytokine IL-10 therapy may be a novel approach to treat atherosclerosis for the future [10]. The elevated IL-17 levels reduced remarkably atherosclerotic plaques, and the IL-17 deficiency accelerated atherosclerotic plaque formation and atherosclerotic plaque instability. Increased levels of antiatherogenic inflammatory cytokine IL-17 were related to the better prognosis in the patients with acute myocardial infarction, supporting the protecting role of IL-17 in coronary artery disease. The patients with a reduction of IL-17 increased the risk for death and recurrent acute myocardial infarction, suggesting a crucial role in antiatherogenic inflammatory effects of IL-17 [11]. IL-13 as antiatherogenic inflammatory cytokine had an atheroprotective effect and played crucial roles in promoting atherosclerotic plaque stability and inhibiting the development and progression of atherogenesis. IL-13 deficiency profoundly promoted atherosclerotic plaque formation and atherosclerotic plaque instability. IL-13 therapy may be a potential novel target for preventing and treating atherosclerosis [12]. Atherosclerosis was the chronic inflammatory process of the arterial intima. IL-37 was a new antiatherogenic inflammatory cytokine and had strong anti-inflammatory properties and was a protective role in atherosclerosis. IL-37 remarkably inhibited the levels of proatherogenic inflammatory cytokines (IL-6, IL-1*β*, and TNF-*α*) and increased the level of antiatherogenic inflammatory cytokine IL-10. IL-37 treatment promoted a remarkable diminution in the atherosclerotic plaque size and atherosclerotic plaque stability. IL-37 treatment may be a new approach for reducing atherosclerosis [16].

Our findings showed that the levels of IL-10, IL-17, IL-13, and IL-37 as antiatherogenic inflammatory cytokines were decreased significantly in the elderly patients with the proximal, middle, and distal coronary artery reocclusions after coronary stent implantations. The decreased levels of IL-10, IL-17, IL-13, and IL-37 were involved in the pathogenesis of the proximal, middle, and distal coronary artery reocclusions in the elderly patients after coronary stent implantations. IL-10 deficiency elevated atherosclerotic plaque size and promoted the development of atherosclerosis, and the human IL-10 gene transfer decreased atherosclerosis and atherosclerotic plaque instability [10]. The elevated IL-17 levels reduced remarkably atherosclerotic plaques, and the IL-17 deficiency accelerated atherosclerotic plaque formation and atherosclerotic plaque instability [11]. IL-13 had an atheroprotective effect and played crucial roles in promoting atherosclerotic plaque stability and inhibiting the development and progression of atherogenesis. IL-13 deficiency profoundly promoted atherosclerotic plaque formation and atherosclerotic plaque instability [12]. IL-37 was a new antiatherogenic inflammatory cytokine and played the strong atheroprotective and anti-inflammatory roles in atherosclerosis. IL-37 therapy reduced remarkably the atherosclerotic plaque size and plaque stability [16]. The present study suggested that the decreased levels of IL-10, IL-17, IL-13, and IL-37 were involved in the proximal, middle, and distal coronary artery reocclusions in the elderly patients after coronary stent implantations. Imbalances between proatherogenic inflammatory and antiatherogenic inflammatory mediators may promote the formation of the proximal, middle, and distal coronary artery reocclusions in elderly patients after coronary stent implantations.

Interventional coronary therapy of coronary chronic total occlusions was a rapidly evolving area, and the prevalence of coronary chronic total occlusions was the highest in elderly people. However, most clinical studies excluded elderly patients, and clinical study data in elderly patients were lacking. Few studies were available regarding outcomes of interventional therapy for coronary chronic total occlusions in elderly patients in the current drug-eluting stent era [27]. There was limited evidence of the differences between the elderly (≥65 years) and younger patients (<65 years) regarding coronary artery reocclusions after coronary stent implantations. We therefore conducted the study in patients with distal coronary artery (Dist CA) reocclusions, middle coronary artery (Mid CA) reocclusions, proximal coronary artery (Prox CA) reocclusions, and proximal+middle coronary artery (Prox+Mid CA) reocclusions to identify differences of degree of coronary artery reocclusions between elderly and younger patients.

The study indicated that for each 10-millimeter increase in distance from the coronary ostium to the culprit lesions, the risk of coronary artery occlusions was remarkably reduced by 13% in the RCA, 30% in the LAD, and 26% in the LCX. The proximal artery occlusions were associated with high mortality [28], and the patients with proximal LAD stenosis had higher mortality and major adverse cardiac events after coronary stenting [29]. Prediction of occlusion of the proximal RCA was very important because proximal RCA occlusion was frequently related to the right ventricular infarction and had a high mortality rate [30, 31]. Proximal LCX occlusion caused extensive injury to the entire posterior myocardial wall, whereas distal LCX occlusion only produced local ischemia in the posteroinferior myocardial wall [32]. The research also showed that proximal coronary artery lesions often diffuse long lesions, whereas distal coronary artery lesions were more often short and discrete coronary lesions [33]. The prevalence of coronary artery occlusions was highest in elderly patients. Few data were available on coronary artery occlusions in very elderly patients [34].

The pathological mechanism of coronary restenosis and coronary reocclusion from human autopsy coronary arteries after coronary stent implantations was that the drug-eluting stent (DES) resulted in coronary reocclusion followed by restenosis due to persistent chronic inflammation after DES implantations [35]. The coronary balloon angioplasty and coronary stenting changed the coronary arterial structure [36] and caused extensive coronary arterial wall injury with greater length and greater tearing of the coronary medial wall. The coronary medial wall disruption was more frequently found in the total coronary reocclusions in patients when compared to the coronary stent restenosis in patients, and the inflammatory responses were related to greater length and greater tearing of the coronary medial wall after coronary balloon angioplasty and coronary stenting. The robust inflammatory responses and infiltration of the coronary arteries were found at all stages of coronary medial injury, and inflammatory responses inhibited the release antirestenosis drugs as DES [36–38] and further promoted the medial thickness of coronary arteries, coronary restenosis, and total coronary reocclusions in patients [39]. Therefore, the pathological mechanism of coronary reocclusion after coronary stent implantations was attributed to the fact that the coronary balloon angioplasty and coronary stenting changed the coronary arterial structure and caused the extensive coronary arterial wall injury in response to inflammatory mediators (IL-1 *β*, IL-6, IL-8, TNF-*α*, and hs-CRP), and the intense inflammatory response simultaneously inhibited the release antirestenosis drugs as DES, leading to the coronary medial thickness and total coronary reocclusions in patients after DES implantations.

The importance of the current research was designed to study the reocclusions of different sites of coronary artery in the elderly patients and the effects of imbalances between proatherogenic inflammatory and antiatherogenic inflammatory mediators on reocclusions of different segments of RCA, LCX, and LAD after coronary artery implantations. The results showed that elderly patients had the proximal, middle, and distal reocclusions of RCA, LCX, and LAD after coronary artery implantations. The study also found that the imbalance between proatherogenic inflammatory and antiatherogenic inflammatory mediators was related to the proximal, middle, and distal reocclusions of RCA, LCX, and LAD and the high levels of proinflammatory cytokines increased the risks of proximal and proximal+middle reocclusions of RCA, LCX, and LAD. Therefore, the elderly patients with the proximal and proximal+middle reocclusions of RCA, LCX, and LAD had higher risks of mortality and major adverse cardiac events after coronary stenting. This study was important for predicting the progression of different segments of the reocclusions in RCA, LCX, and LAD.

It was clinically important to recognize the elderly patients with proximal, middle, and distal coronary artery reocclusions, which can result in higher mortality and major adverse cardiac events. Such assessment should be simple, quick, and noninvasive detection and prediction of severity of coronary artery reocclusions after coronary stent implantations. We think that the determinations of circulating levels of proatherogenic inflammatory and antiatherogenic inflammatory mediators may be an efficient method for clinical decision making in coronary artery reocclusions of elderly patients after coronary stent implantations. Our present study reveals that IL-1 *β*, IL-6, IL-8, TNF-*α*, hs-CRP, IL-10, IL-17, IL-13, and IL-37 are the independent predictors for coronary artery reocclusions of elderly patients after coronary stent implantations. According to these data, a strategy that protects proximal, middle, and distal reocclusions from RCA, LCX, and LAD may be effective for preventing coronary artery reocclusions after coronary stent implantations. Furthermore, appropriate anti-inflammatory cytokine therapy should be considered to prevent the coronary artery reocclusions in elderly patients after coronary stent implantations. Early identification of high-risk patients with proximal, middle, and distal reocclusions will lead to future advances in proximal, middle, and distal reocclusion detection technology and potentially preventive strategies for proximal, middle, and distal reocclusions. Moreover, the present study raises the possibility of designing standard or modified drug-eluting stents to prevent proximal, middle, and distal reocclusions in elderly patients after coronary stent implantations.

## 5. Conclusion

The present study showed that the increased levels of proatherogenic inflammatory mediators and the decreased levels of antiatherogenic inflammatory cytokines were associated with the proximal, middle, and distal coronary artery reocclusions in the elderly patients after coronary stent implantations, and the imbalances between proatherogenic inflammatory and antiatherogenic inflammatory mediators may further accelerate the formation of the proximal, middle, and distal reocclusions coronary artery reocclusions in the elderly patients after coronary stent implantations.

## Figures and Tables

**Table 1:** Baseline characteristics of elderly patients with the proximal, middle, and distal coronary artery reocclusions.

	CON *n* = 69	Dist CA reocclusions *n* = 165	Mid CA reocclusions *n* = 147	Prox CA reocclusions *n* = 123	Prox+Mid CA reocclusions *n* = 101
Gender (male/female)	39/30	85/80	77/70	63/60	50/51
65-70-year-old age	13/10	20/16	19/21	20/18	12/15
71-76-year-old age	11/8	23/14	15/20	17/15	10/17
77-82-year-old age	9/7	20/26	25/12	14/12	11/9
83-88-year-old age	6/5	22/24	18/17	12/15	17/10
Familial coronary artery disease	0/0	41/37	64/57	54/50	51/48
Dyslipidaemia	0/0	74/63	59/47	54/51	49/42
Chest pain	0/0	62/54	63/59	51/49	51/45
Diabetes mellitus	0/0	59/61	57/50	48/45	47/41
Hypertension	0/0	70/69	49/52	39/41	41/46
Alcohol consumption	0/0	54/43	35/30	40/34	40/49
Smoker	0/0	67/58	61/57	63/50	52/45

Data were expressed as the number of male and female subjects.

**Table 2:** Cytokine and hs-CRP levels in the elderly patients with the proximal, middle, and distal CA reocclusions.

	CON *n* = 69	Dist CA reocclusions *n* = 165	Mid CA reocclusions *n* = 147	Prox CA reocclusions *n* = 123	Prox+Mid CA reocclusions *n* = 101
IL-1 *β* (pg/mL)	51.0 ± 8.2	79.5 ± 11.0^∗^	86.4 ± 12.6^∗∗^	141.7 ± 14.5^∗∗∗^	173.2 ± 23.0^∗∗∗∗^
IL-6 (pg/mL)	81.2 ± 20.4	97.4 ± 21.8^∗^	110.9 ± 40.5^∗∗^	130.0 ± 62.7^∗∗∗^	164.5 ± 76.9^∗∗∗∗^
IL-8 (pg/mL)	4.0 ± 0.9	6.0 ± 1.3^∗^	9.6 ± 4.1^∗∗^	11.7 ± 7.0^∗∗∗^	13.8 ± 9.4^∗∗∗∗^
TNF-*α* (ng/L)	60.3 ± 13.5	74.8 ± 14.7^∗^	89.5 ± 16.3^∗∗^	110.0 ± 18.6^∗∗∗^	138.9 ± 21.0^∗∗∗∗^
IL-10 (pg/mL)	19.1 ± 10.3	16.9 ± 8.2^∗^	13.7 ± 5.0^∗∗^	10.1 ± 3.1^∗∗∗^	7.2 ± 1.3^∗∗∗∗^
IL-17 (pg/mL)	15.3 ± 5.6	13.0 ± 4.3^∗^	11.9 ± 3.8^∗∗^	7.9 ± 2.4^∗∗∗^	6.1 ± 1.6^∗∗∗∗^
IL-13 (pg/mL)	410.1 ± 85.0	360.8 ± 65.2^∗^	278.5 ± 46.0^∗∗^	260.1 ± 40.8^∗∗∗^	247.3 ± 29.8^∗∗∗∗^
IL-37 (pg/mL)	124.0 ± 15.3	113.0 ± 13.7^∗^	89.3 ± 11.5^∗∗^	71.1 ± 9.5^∗∗∗^	56.8 ± 6.4^∗∗∗∗^
hs-CRP (mg/L)	3.2 ± 0.7	4.3 ± 0.8^∗^	7.1 ± 1.4^∗∗^	10.2 ± 2.2^∗∗∗^	14.0 ± 2.8^∗∗∗∗^

Student's *t*-test:^∗^*P* < 0.001 (CON group/Dist CA reocclusion group). ^∗∗^*P* < 0.001 (Dist CA reocclusion group/Mid CA reocclusion group). ^∗∗∗^*P* < 0.001 (Mid CA reocclusion group/Prox CA reocclusion group). ^∗∗∗∗^*P* < 0.001 (Prox CA reocclusion group/Prox+Mid CA reocclusion group). Group comparisons (CON/Dist CA reocclusion group/Mid CA reocclusion group/Prox CA reocclusion group/Prox+Mid CA reocclusion group) were made using ANOVA, *P* < 0.001.

**Table 3:** Cytokine and hs-CRP levels in the elderly patients with the proximal, middle, and distal RCA reocclusions.

	CON *n* = 69	Dist RCA reocclusions *n* = 60	Mid RCA reocclusions *n* = 53	Prox RCA reocclusions *n* = 49	Prox+Mid RCA reocclusions *n* = 40
IL-1 *β* (pg/mL)	51.0 ± 8.2	59.7 ± 9.0^∗^	67.2 ± 11.5^∗∗^	81.1 ± 14.3^∗∗∗^	107.3 ± 21.7^∗∗∗∗^
IL-6 (pg/mL)	81.2 ± 20.4	93.1 ± 28.7^∗^	109.2 ± 39.4^∗∗^	120.6 ± 45.0^∗∗∗^	142.3 ± 51.6^∗∗∗∗^
IL-8 (pg/mL)	4.0 ± 0.9	5.2 ± 1.0^∗^	6.9 ± 2.5^∗∗^	8.3 ± 4.1^∗∗∗^	11.0 ± 6.3^∗∗∗∗^
TNF-*α* (ng/L)	60.3 ± 13.5	79.6 ± 14.8^∗^	95.3 ± 16.2^∗∗^	113.7 ± 19.0^∗∗∗^	130.8 ± 21.9^∗∗∗∗^
IL-10 (pg/mL)	19.1 ± 10.3	15.0 ± 7.1^∗^	11.4 ± 5.1^∗∗^	7.3 ± 3.4^∗∗∗^	3.8 ± 1.2^∗∗∗∗^
IL-17 (pg/mL)	15.3 ± 5.6	10.1 ± 4.9^∗^	8.0 ± 3.2^∗∗^	5.1 ± 1.1^∗∗∗^	3.0 ± 0.8^∗∗∗∗^
IL-13 (pg/mL)	410.1 ± 85.0	301.5 ± 77.0^∗^	219.6 ± 59.7^∗∗^	130.4 ± 34.9^∗∗∗^	110.1 ± 23.5^∗∗∗∗^
IL-37 (pg/mL)	124.0 ± 15.3	110.3 ± 12.0^∗^	97.6 ± 10.1^∗∗^	70.5 ± 8.2^∗∗∗^	51.7 ± 5.0^∗∗∗∗^
hs-CRP (mg/L)	3.2 ± 0.7	3.9 ± 0.7^∗^	6.0 ± 1.1^∗∗^	7.9 ± 1.5^∗∗∗^	10.3 ± 1.9^∗∗∗∗^

Student's *t*-test: ^∗^*P* < 0.001 (CON group/Dist RCA reocclusion group). ^∗∗^*P* < 0.001 (Dist RCA reocclusion group/Mid RCA reocclusion group). ^∗∗∗^*P* < 0.001 (Mid RCA reocclusion group/Prox RCA reocclusion group). ^∗∗∗∗^*P* < 0.001 (Prox RCA reocclusion group/Prox+Mid RCA reocclusion group). Group comparisons (CON/Dist RCA reocclusion group/Mid RCA reocclusion group/Prox RCA reocclusion group/Prox+Mid RCA reocclusion group) were made using ANOVA, *P* < 0.001.

**Table 4:** Cytokine and hs-CRP levels in the elderly patients with the proximal, middle, and distal LCX reocclusions.

	CON *n* = 69	Dist LCX reocclusions *n* = 50	Mid LCX reocclusions *n* = 45	Prox LCX reocclusions *n* = 39	Prox+Mid LCX reocclusions *n* = 32
IL-1 *β* (pg/mL)	51.0 ± 8.2	63.9 ± 8.5^∗^	79.6 ± 10.0^∗∗^	130.7 ± 13.1^∗∗∗^	160.5 ± 21.3^∗∗∗∗^
IL-6 (pg/mL)	81.2 ± 20.4	101.3 ± 30.1^∗^	130.2 ± 39.3^∗∗^	172.8 ± 45.0^∗∗∗^	209.1 ± 54.2^∗∗∗∗^
IL-8 (pg/mL)	4.0 ± 0.9	5.9 ± 1.3^∗^	8.3 ± 3.1^∗∗^	10.1 ± 5.7^∗∗∗^	15.7 ± 7.4^∗∗∗∗^
TNF-*α* (ng/L)	60.3 ± 13.5	79.4 ± 15.2^∗^	91.6 ± 17.0^∗∗^	130.3 ± 21.8^∗∗∗^	169.2 ± 30.5^∗∗∗∗^
IL-10 (pg/mL)	19.1 ± 10.3	11.0 ± 9.1^∗^	8.3 ± 6.2^∗∗^	5.5 ± 3.0^∗∗∗^	2.9 ± 1.2^∗∗∗∗^
IL-17 (pg/mL)	15.3 ± 5.6	10.1 ± 4.9^∗^	7.6 ± 3.0^∗∗^	5.0 ± 2.8^∗∗∗^	3.1 ± 1.0^∗∗∗∗^
IL-13 (pg/mL)	410.1 ± 85.0	310.0 ± 79.3^∗^	209.8 ± 50.1^∗∗^	90.4 ± 31.0^∗∗∗^	70.9 ± 20.6^∗∗∗∗^
IL-37 (pg/mL)	124.0 ± 15.3	112.5 ± 12.4^∗^	97.0 ± 10.4^∗∗^	64.8 ± 7.2^4^	53.5 ± 5.1^∗∗∗∗^
hs-CRP (mg/L)	3.2 ± 0.7	4.1 ± 0.8^∗^	6.9 ± 1.3^∗∗^	8.2 ± 1.6^∗∗∗^	11.4 ± 2.1^∗∗∗∗^

Student's *t*-test: ^∗^*P* < 0.001 (CON group/Dist LCX reocclusion group). ^∗∗^*P* < 0.001 (Dist LCX reocclusion group/Mid LCX reocclusion group). ^∗∗∗^*P* < 0.001 (Mid LCX reocclusion group/Prox LCX reocclusion group). ^∗∗∗∗^*P* < 0.001 (Prox LCX reocclusion group/Prox+Mid LCX reocclusion group). Group comparisons (CON/Dist LCX reocclusion group/Mid LCX reocclusion group/Prox LCX reocclusion group/Prox+Mid LCX reocclusion group) were made using ANOVA, *P* < 0.001.

**Table 5:** Cytokine and hs-CRP levels in the elderly patients with the proximal, middle, and distal LAD reocclusions.

	CON *n* = 69	Dist LAD reocclusions *n* = 55	Mid LAD reocclusions *n* = 49	Prox LAD reocclusions *n* = 35	Prox+Mid LAD reocclusions *n* = 29
IL-1 *β* (pg/mL)	51.0 ± 8.2	69.0 ± 8.9^∗^	77.3 ± 10.5^∗∗^	109.8 ± 23.1^∗∗∗^	137.2 ± 27.4^∗∗∗∗^
IL-6 (pg/mL)	81.2 ± 20.4	93.4 ± 29.0^∗^	131.8 ± 42.3^∗∗^	147.9 ± 49.2^∗∗∗^	160.5 ± 57.3^∗∗∗∗^
IL-8 (pg/mL)	4.0 ± 0.9	6.1 ± 1.1^∗^	8.0 ± 3.2^∗∗^	13.5 ± 5.0^∗∗∗^	17.0 ± 7.5^∗∗∗∗^
TNF-*α* (ng/L)	60.3 ± 13.5	72.0 ± 14.7^∗^	90.6 ± 16.0^∗∗^	118.4 ± 18.2^∗∗∗^	125.0 ± 23.1^∗∗∗∗^
IL-10 (pg/mL)	19.1 ± 10.3	14.1 ± 9.0^∗^	9.5 ± 7.2^∗∗^	5.0 ± 3.2^∗∗∗^	2.9 ± 1.1^∗∗∗∗^
IL-17 (pg/mL)	15.3 ± 5.6	10.1 ± 5.0^∗^	6.9 ± 3.0^∗∗^	3.6 ± 3.7^∗∗∗^	1.8 ± 0.9^∗∗∗∗^
IL-13 (pg/mL)	410.1 ± 85.0	381.6 ± 77.4^∗^	311.2 ± 65.7^∗∗^	285.0 ± 43.1^∗∗∗^	199.7 ± 20.8^∗∗∗∗^
IL-37 (pg/mL)	124.0 ± 15.3	109.4 ± 13.0^∗^	97.3 ± 11.4^∗∗^	81.9 ± 9.7^∗∗∗^	59.8 ± 6.9^∗∗∗∗^
hs-CRP (mg/L)	3.2 ± 0.7	5.3 ± 1.0^∗^	7.5 ± 1.5^∗∗^	10.7 ± 2.0^∗∗∗^	12.5 ± 2.6^∗∗∗∗^

Student's *t*-test: ^∗^*P* < 0.001 (CON group/Dist LAD reocclusion group). ^∗∗^*P* < 0.001 (Dist LAD reocclusion group/Mid LAD reocclusion group). ^∗∗∗^*P* < 0.001 (Mid LAD reocclusion group/Prox LAD reocclusion group). ^∗∗∗∗^*P* < 0.001 (Prox LAD reocclusion group/Prox+Mid LAD reocclusion group). Group comparisons (CON/Dist LAD reocclusion group/Mid LAD reocclusion group/Prox LAD reocclusion group/Prox+Mid LAD reocclusion group) were made using ANOVA, *P* < 0.001.

**Table 6:** The related data in the length and diameter of stents.

	Dist CA reocclusions *n* = 165	Mid CA reocclusions *n* = 147	Prox CA reocclusions *n* = 123	Prox+Mid CA reocclusions *n* = 101
Stent lengths				
8.0 mm (*n* (%))	48 (29)	30 (20)	32 (26)	21 (21)
15.0 mm (*n* (%))	46 (28)	41 (28)	31 (25)	25 (25)
18.0 mm (*n* (%))	45 (27)	42 (29)	35 (28)	23 (23)
23.0 mm (*n* (%))	26 (16)	33 (22)	25 (20)	32 (31)
*P*	0.14	0.85	0.43	0.16

Stent diameters				
2.50 mm (*n* (%))	48 (29)	30 (20)	32 (26)	21 (21)
3.00 mm (*n* (%))	46 (28)	41 (28)	31 (25)	25 (25)
3.50 mm (*n* (%))	45 (27)	42 (29)	35 (28)	23 (23)
4.00 mm (*n* (%))	26 (16)	33 (22)	25 (20)	32 (31)
*P*	0.14	0.85	0.43	0.16

**Table 7:** Relevant data of patients with main clinical symptoms and evidence of myocardial ischemia.

	Dist CA reocclusions *n* = 165	Mid CA reocclusions *n* = 147	Prox CA reocclusions *n* = 123	Prox+Mid CA reocclusions *n* = 101
TIMI				
Grade 0 (*n* (%))	165 (100)	147 (100)	123 (26)	101 (100)

SAP				
Class I (*n* (%))	61 (37)	55 (37)	34 (28)	21 (21)
Class II (*n* (%))	64 (39)	54 (36)	44 (35)	35 (35)
Class III (*n* (%))	40 (24)	38 (26)	45 (37)	45 (44)
*P*	0.41	0.28	0.19	0.06

UAP				
Grade-I	74 (45)	67 (45)	26 (21)	21 (21)
Grade-II	46 (28)	35 (24)	46 (37)	35 (35)
Grade-III	45 (27)	45 (31)	51 (41)	45 (45)
*P*	0.30	0.54	0.21	0.06

**Table 8:** Atorvastatin therapy and levels of blood lipids in patients with coronary artery reocclusion.

Lipids (mg/dL)	CON *n* = 69	Dist CA reocclusions *n* = 165	Mid CA reocclusions *n* = 147	Prox CA reocclusions *n* = 123	Prox+Mid CA reocclusions *n* = 101
TC	173.3 ± 32.6	165.4 ± 32.4	180.1 ± 32.4	178.8 ± 34.0	183.4 ± 36.6
TG	124.5 ± 22.3	130.6 ± 20.1	120.5 ± 23.8	121.1 ± 24.9	131.7 ± 25.3
HDL-C	44.0 ± 7.2	45.3 ± 8.4	47.3 ± 9.0	40.3 ± 8.0	46.2 ± 9.1
LDL-C	122.6 ± 23.1	121.7 ± 21.3	129.6 ± 24.9	125.8 ± 25.1	123.9 ± 24.7
VLDL-C	24.8 ± 3.4	23.1 ± 3.6	25.3 ± 4.0	27.9 ± 5.3	28.0 ± 5.8

Student's *t*-test *P* > 0.05 (CON group/Dist CA reocclusion group). *P* > 0.05 (Dist CA reocclusion group/Mid CA reocclusion group). *P* > 0.05 (Mid CA reocclusion group/Prox CA reocclusion group). *P* > 0.05 (Prox CA reocclusion group/Prox+Mid CA reocclusion group). Group comparisons (CON/Dist CA reocclusion group/Mid CA reocclusion group/Prox CA reocclusion group/Prox+Mid CA reocclusion group) were made using ANOVA, *P* > 0.05.

**Table 9:** Multivariate regression analysis to evaluate the significance of variables for the proximal, middle, and distal coronary artery reocclusions.

Variables	Odds ratio	95% CI	*P* value
Age	1.27	0.24-1.84	0.57
Gender	2.63	0.74-3.24	0.14
Familial coronary artery disease	1.32	0.81-1.07	0.49
Dyslipidaemia	2.34	0.26-6.24	0.09
Chest pain	1.16	0.63-11.6	0.12
Diabetes mellitus	1.41	0.71-2.35	0.41
Hypertension	3.70	0.83-15.07	0.10
Alcohol consumption	5.62	0.91-25.04	0.08
Smoking	3.25	0.48-17.31	0.50
Angiography	4.19	0.53-20.32	0.36
IL-1 *β*	3.15	1.27-7.61	0.02
IL-6	2.04	1.19-1.92	0.03
IL-8	3.51	1.37-3.52	0.01
TNF-*α*	4.89	1.68-17.1	0.003
IL-10	1.36	1.24-2.15	0.02
IL-17	1.57	1.46-1.98	0.01
IL-13	5.63	1.38-7.63	0.02
IL-37	4.31	1.22-3.41	0.04

## Data Availability

The data used to support the findings of this study are available from the corresponding author upon request.

## Abbreviations

IL-1 *β*: Interleukin-1 *β*
IL-6: Interleukin-6
IL-8: Interleukin-8
TNF-*α*: Tumor necrosis factor-*α*
IL-10: Interleukin-10
IL-17: Interleukin-17
IL-13: Interleukin-13
IL-37: Interleukin-37
Dist CA: Distal coronary artery
Mid CA: Middle coronary artery
Prox CA: Proximal coronary artery
CON: Control
Dist RCA: Distal right coronary artery
Mid RCA: Middle right coronary artery
Prox RCA: Proximal right coronary artery
Dist LCX: Distal left circumflex artery
Mid LCX: Middle left circumflex artery
Prox LCX: Proximal left circumflex artery
Dist LAD: Distal left anterior descending artery
Mid LAD: Middle left anterior descending artery
Prox LAD: Proximal left anterior descending artery
TIMI: Thrombolysis in myocardial ischemia
SAP: Stable angina pectoris
UAP: Unstable angina pectoris
TC: Total cholesterol
TG: Triglyceride
HDL-C: High-density lipoprotein cholesterol
LDL-C: Low-density lipoprotein-cholesterol
VLDL-C: Very low-density lipoprotein-cholesterol
hs-CRP: High sensitive C reaction protein
DES: Drug-eluting stent.
